# *“The system here isn’t on patients’ side”-* perspectives of women and men on the barriers to accessing and utilizing maternal healthcare services in South Sudan

**DOI:** 10.1186/s12913-017-2788-9

**Published:** 2018-01-09

**Authors:** Ngatho S. Mugo, Michael J. Dibley, Eliaba Yona Damundu, Ashraful Alam

**Affiliations:** 1 Sydney School of Public Health, Edward Ford Building (A27), University of Sydney, Sydney, NSW 2006 Australia; 2 UNICEF South Sudan, Toto Chan Compound, P.O. Box 45, Juba South, Sudan

**Keywords:** Barriers, Maternal health care, Perceptions, Qualitative research, South Sudan

## Abstract

**Background:**

In fragile and war-affected setting such as South Sudan, a combination of physical environmental, socioeconomic factors and healthcare’s characteristic contributes to higher rates of home delivery attended by unskilled attendants. This study aims to understand the community members’ experience, perceptions and the barriers in relation to accessing and utilizing maternal healthcare services in South Sudan.

**Methods:**

We conducted in-depth one-on-one interview with 30 women and 15 men to investigate their perspectives on the barriers to access maternal and child health related services. We purposively selected women and their partners in this study.

**Results:**

Our study revealed that inadequate quality of antenatal care services such as lack of essential medicine, supplies and tools was linked to individual’s mothers dissatisfaction with the services they received. In addition, sudden onset of labor and lack of safety and security were important reasons for home delivery in this study. Furthermore, lack of transport as a result of a combination of long distance to a facility and associated costs either restricted or delayed women reaching the health facilities.

**Conclusions:**

Our study highlighted an urgent need for the government of South Sudan to implement security and safety measures in order to improved access to delivery service at night. Incorporating private transports to provide access to affordable and reliable transport services for pregnant and post-partum women is also important. Increasing the budget allocation for medicine and health supplies and improving management of medicine and supply chain logistics are essential.

## Background

The reduction of the maternal mortality ratio to less than 70 per 100,000 live births between 2016 and 2030 is one of the global priority targets of the Sustainable Development Goals [1]. Globally, during the millennium development goals era the proportion of deliveries attended by a skilled attendant increased from 59 to 71% from 1990 to 2015 [2]. Yet more than one in four newborns and their mothers still have no access to essential medical care during childbirth [2]. Evidence suggests positive associations between access to facility-based services from skilled birth attendants during pregnancy, delivery and post-delivery, and improved maternal health outcomes [3–7]. However, in a setting affected by conflicts, displacement, and natural disasters, access to such services is very limited and the risk of death following pregnancy and childbirth is high [8, 9].

South Sudan is a fragile and war-affected setting that gained independence in 2011 [10]. Since then, the country has experienced internal arm conflict, political instability, insecurity and the closure or destruction of the healthcare facility. In 2015 South Sudan was among the developing countries with the highest maternal mortality rate and it was estimated at 789 per 100,000 live birth [9]. The lifetime risk of maternal mortality from maternal causes in South Sudan is still very high at 1 in 50 deaths in 2015 compared to 1 in 180 in developing countries verses 1 in 4900 in developed countries [9, 11]. A combination of physical environment, social and economic factors, and the individual woman’s characteristics and behaviors increases the lifetime risk of maternal mortality among women of reproductive age and among their under-five children. According to 2010 South Sudan household survey report the neonatal mortality rate was estimated at 40 per 1000 live birth, an infant mortality rate of 74 per 1000 live birth and under-five mortality rates of 101 per 1000 live birth [12, 13].

In response to higher maternal and under-five mortality, the government of South Sudan made a commitment to implement free access to maternity and child healthcare services [14, 15]. However, the decades of the civil war have severely contributed to collapse of the public health system. Currently Non-Governmental Organizations (NGOs) provided approximately 80% of the basic healthcare services [16]. These services are provided though facilities based clinical care. The primary health care unit is the immediate point of contact for antenatal and postnatal care services and provide basic preventive and curative services. In addition to the services offered by health care units, Primary Health Care Centers offer basic diagnostic laboratory services and maternity care [17]. County and State Hospitals provide secondary care including comprehensive obstetric care, in-patient care and surgery [17–19].

**Fig. 1:**
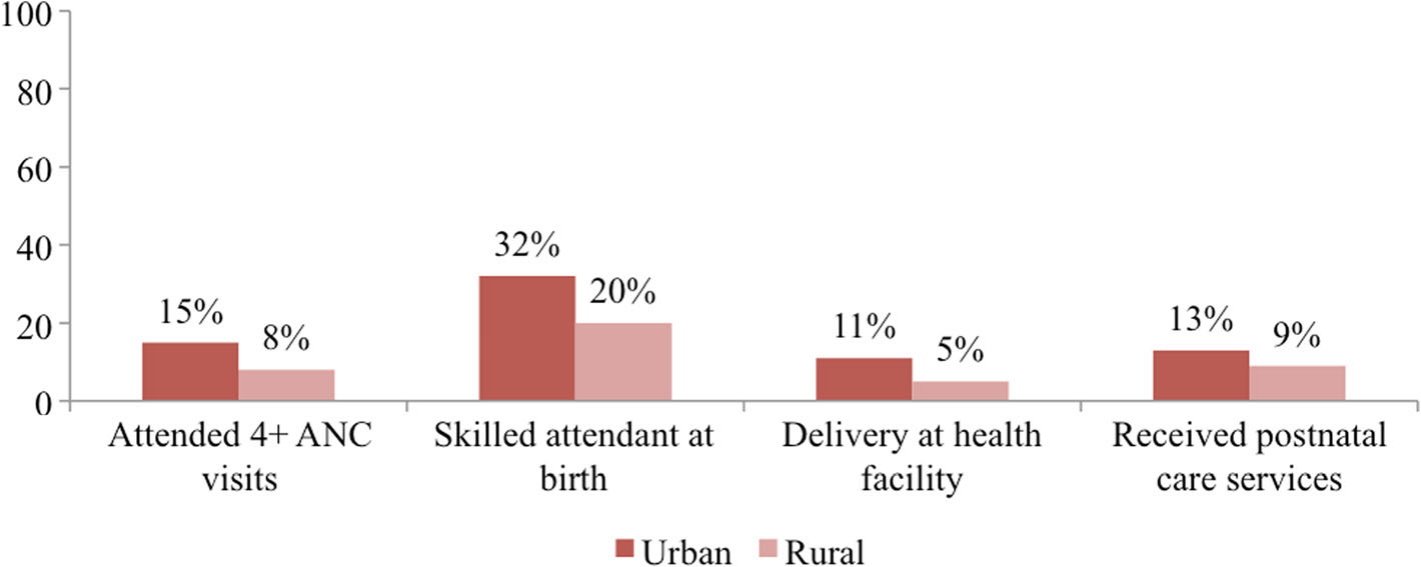
Inequalities in coverage of maternal healthcare services by place of residence ** Source: South Sudan household survey 2010

**Fig. 2:**
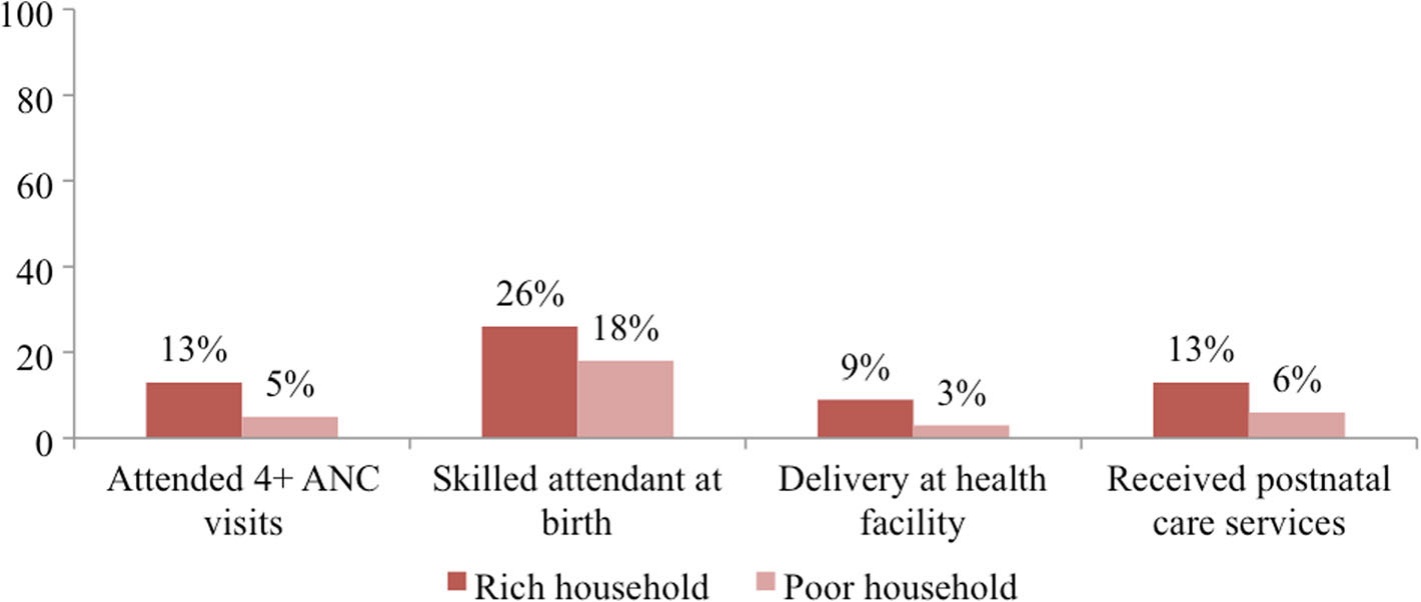
Inequalities in coverage of maternal healthcare services by household wealth** Source: South Sudan household survey 2010

Yet coverage of maternal health care services is generally very low with high levels of inequalities in South Sudan (see Figs. 1 and 2). As a result only 19% of deliveries take place at a facility as oppose to eight in ten births (81%) taking place at home [13]. In order to prevent maternal mortality following pregnancy, delivery and post-delivery in South Sudan, there is an urgent need to understand the reasons for low utilization of maternal and child health services.

Previous analysis of South Sudan household survey data identified factors associated with non-use of maternal health care services and health facilities for delivery [20–22]. However, these studies did not address the factors the South Sudanese women experience in accessing maternal and child health services. This study aims to understand the community members’ perceptions and experience of and barriers they face to use maternal health services.

## Methods

### Study sites and sampling

This analysis was part of a wider study conducted in early December 2015 to end of January 2016 in Juba county central Equatoria State. We purposively selected health services located in Juba town, Kator, and Munuki for security and accessibility reasons. The wider study aimed to explore the perceived and experienced barriers faced by the community members to access healthcare services, as well as the barriers faced by the healthcare providers to deliver healthcare services to their client.

The current paper reports the findings generated from the women and their husbands on perceived and experienced barriers to receiving maternal health services at the Juba Teaching Hospital, the Juba Military Hospital, and the Nykory Primary Health Care Center. Our study participants consisted of 30 mothers, 10 in each type of health care facility, with children aged less than 3 months, who had given birth either at home or in a health facility, and 15 husbands (5 in each health care facility).

### Data collection

Prior to data collection we recruited and trained one research assistant in qualitative data collection methods. We conducted in-depth one-on-one interviews firstly with all mothers then secondly we cross check the information with their husbands to investigate each individual’s perspectives on the barriers to access to maternal and child health related services. Interviews with women were conducted in a confidential place mostly on the premises of the health facility at each of the selected health facility for their convenience and ease accessibility. Interviews with husbands were conducted at the Juba Staff Club. To recruit the husbands, firstly we purposively selected eligible women who were willing to participate in the study. The husbands who were accompanying their wife were invited to participate and the consented husbands were interviewed. If the woman was alone or accompanied by anyone other than her husband, we collected the husband’s contact details to make an attempt to contact him. Through this process we finally interviewed 15 husbands. All interviews were conducted in local South Sudan Arabic. We developed and used separate guidelines to administer the in-depth interviews with each type of respondent. All the interviews were audio recorded.

### Data analysis

We followed multiple steps to analyze the data. First, the lead researcher (NSM) transcribed verbatim each audio-recorded interview conducted in the local language and then translated the interview into English and saved it as Microsoft Word document. Second, a team of two researchers crosschecked the translation against the audio recording and the transcription. Third, NSM prepared a draft code list by carefully reading two transcripts, which were independently checked by another researcher (AA). Subsequently NSM and AA discussed the draft code list and developed a code list. NSM then manually coded all transcripts. As the study was explorative and descriptive in nature, we applied an inductive coding procedure where themes were derived from the data that were related to our research questions [23]. Fourth, the data were organized and compiled into separate files based on each thematic code. Fifth, involved the development of themes, which were classified according to the objective of the study. We applied an inductive thematic approach for data analysis [24]. The analysis team discussed the text pertaining to each thematic code. After several discussions these were consolidated and summarised in a document for each theme with relevant quotes and text tables. At the end we performed a triangulation of data to compare different responses from mothers and their spouses [25].

### Conceptual framework

**Fig. 3:**
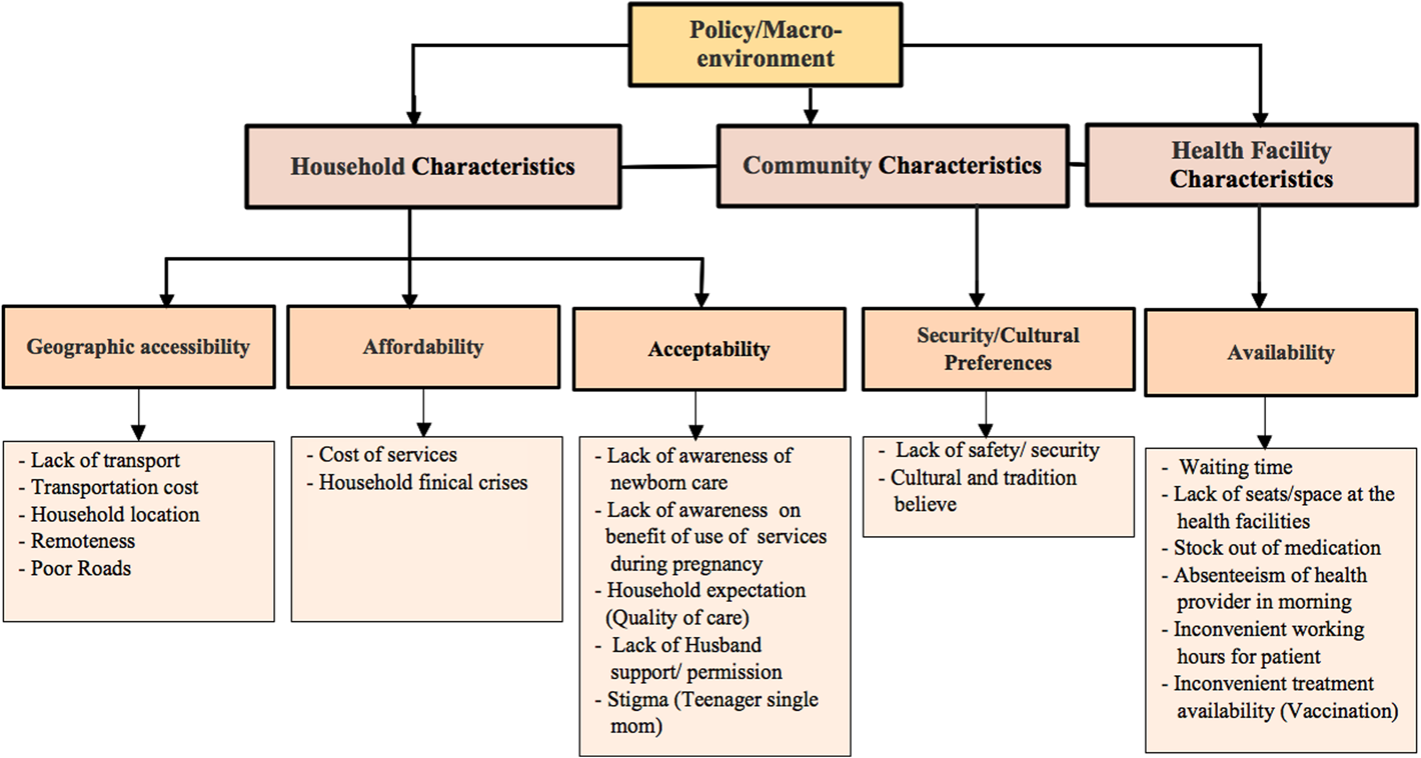
Perceptions of community members on the challenges faced by woman to access and utilize health service. This framework was developed from Peter at el framework

We modified and used the framework developed by Peter at el [26] as a guide to examine and group the key challenges and barriers facing women to access health care services in South Sudan. Figure 3 presents the main challenges identified in this study. According to the framework the distal factors such as policy and or macro-environment have direct effects on the household, community and health facility. In turn, the characteristics of the health facility and the household and community affect the individual mother’s use or nonuse of the services. We classified the challenges into five main categories including: 1) geographical accessibility (the impact of distance and transport to maternal health care services); 2) availability (having access to appropriate types of care to the women who need them); 3) affordability (capacity and willingness to pay for the services); 4) acceptability (the response of health care providers to the social and cultural expectation of the community and the women attending their services); and 5) Security factors (the impact of safety and security instability on community access to healthcare services).

## Result

Table 1 describes the socio-demographic characteristics of the female participants. The majority of the mothers had just had their first child, were unemployed, and had never attended school. Most of the mothers traveled long distances by public transport for 1–2 h to the nearest health facility and had planned for facility delivery.

**Table 1:** Socio-demographic characteristics of women, in depth interview (*n* = 30 women)

Socio-demographic	*N*
*Age*	
20–29	20
30–49	10
*Maternal Education*	
No education	19
Primary and above education	11
*Employment*	
Yes	5
No	25
*Birth order*	
1st -2nd birth	16
3rd birth and over	14
*Distance to near by health facility (by public transport)*	
< Hours	9
> Hours	21
*Attended antenatal care visits*	
Yes	30
No	0
*Birth preparation*	
Yes	25
No	5
*Birth preference*	
Hospital	30
Home	0
*Place of delivery*	
Hospital	10
Home	20

### Household characteristics

#### Geographical accessibility

All of the men participated in this study perceived poor condition of the roads, remoteness, lack of transport, and long distance to the nearest health facilities as the major obstacles for women reaching a health facility for delivery, or when they developed complications.

*“In this area, most of women do deliver at home not because of their intention to deliver here (home) but because it’s the easiest option for them.”* Male participant.

In addition, many women mentioned that delivery at a facility was their first preference, however, their labor happened at night and transport was an issue to reaching the health facility.

*“My labor started at midnight and I gave birth in early morning at home. There was no way that I could make it to Juba teaching hospital since there is no public transport neither any type of transport at night time.” Female participant.*

Several women were alone at home during the labor and felt unable to make it to hospital since distance was an issue and they could not find someone to help them to get to hospital.

*“I was alone at home and then I sensed that the baby is about to come out. I did ask for help form an elder woman who lived nearby. She’s not a ‘Dia’ (midwife) but I did not have any other option.” Female participant.*

#### Affordability

##### User fees

All the mothers perceived paying a small fee reasonable (i.e. about 10 South Sudanese pound (SSP) equivalent to (0.14 USD) for antenatal card and around 5 SSP equivalent to (0.071 USD) for each visit as well as for a pathology test). However, in practice, restricted antenatal services due to financial constraint were also reported.

*“I only attended one antenatal care services during my pregnancy. Everything here (at hospital) is at the cost and we are suffering financially. The little (money) we have is to mange to buy some food.” Female participant.*

Men in this study perceived user fees as responsible for delayed women’s access to care when women experienced complications such as prolong labor, excessive vaginal bleeding, or any other complication of delivery and post delivery. Men, who were financially responsible for the family, were burdened by expenses for transportation, specialists and medicines to treat their wife with complications. The medical center was perceived to have a limited capacity for treatment and would refer patients to higher levels of care in case of complications. This increased the financial burden of men.

*“My wife developed pregnancy complications (postpartum hemorrhage) and it cost me a lot of money to see a gynecology and also for transport. Many family cannot afford the cost and therefore it become an obstacle to access health services for (maternal) complications and saving mothers’ lives.” Male participant.*

Most of the families were affected by the country’s financial crises. Men perceived reduced income and irregular salary disbursement as a major cause of home delivery since families often cannot afford transportation and hospital costs.

#### Acceptability

##### Quality of services

Our study revealed inadequate quality of maternal healthcare as a major constrain for accessing public health services. The quality of public facilities was described as poor, inconvenient and managed by unqualified healthcare providers such as trained- traditional birth attendance (trained-TBAs).

*“In my understanding* government facility *is not well equipped for pregnant women to follow-up there. Also women will not receive adequate services they are expecting and know the sex of the baby since they do not have ultra sound. In case of complication this is a real problem since a specialist is not available and trained-TBAs or midwifery practice are very limited.”* Male participant.

##### Sudden onset of labor

Men and women in this study mentioned that hospital delivery was the first preference in their planning. However, sudden onset of labor and labor starting at night were the major reasons perceived for home delivery.

*“I went for all my antenatal care visits and I gave birth at home. It happens at night and I had no severer pain. I thought its only the beginning but when I was ready to go to hospital it was time and the babe was about to come out, then I have to deliver at home.” Female participant.*

### Community factors

#### Community and cultural preferences and norms

Mothers and their husbands highlighted the impact of tradition on pregnancy and delivery outcomes. Men in this study mentioned that pregnant women were discouraged to eat some type of foods such as eggs, white cheese and cheese product since it was perceived by the community to induce pregnancy and delivery complication such as high blood pressure, preeclampsia or even swollen of the feats or giving birth to a big babe.

*“Pregnant woman are discouraged to eat the food that will make her fat such as eggs because once the women put on weight it also means the baby will put on weight and it will be very difficult for the mother to deliver the baby.” Male participant.*

Lack of husband’s support, lack of women’s autonomy in decision-making concerning use of maternal healthcare services and stigma on unmarried teenage pregnancy were perceived as barriers for accessing medical care. In addition, mothers’ attitude and beliefs were perceived as a barrier to facility delivery in this study. Men also perceived home delivery as safe in the rural community and women were convinced to deliver at their home.

*“In my understanding, if a woman is healthy during pregnancy, she can give birth at home without any issues but the women who are ill are the ones who can developed some problem during delivery.” Female participant.*

#### Security factors

Both women and men in this study perceived insecurity and lack of safety at night as a major reason for home delivery.

*“My labor happened at 10PM. In this area no one will risk his/her live to come out at night because you can lose your life so easily if you meet with those robberies. In spite of the danger, my family managed to bring trained-TBAs whom we know and then I gave birth at home.” Female participant.*

### Health services characteristics

#### Availability

##### Stock out of medication

Many participants, specifically men, perceived the health facilities as lacking essential resources including medicines, childhood vaccines and logistics such as refrigerators to maintain the cold chain.

The stock-out of the medication was the most common factor perceived by mothers during in-depth interviews and was associated with dissatisfaction with use of antenatal care services.

*“Most of the time, there is no enough medicine and after long waiting time we are asked to come back next day. It’s hard for me since I am unwell and too weak to do that. If I had money, I would have bought these medications from the private pharmacy.” Female participant.*

##### Long waiting due to lack of staff

Women were also discouraged by the health system failure to timely attend their needs. Long waiting times at the health facility were perceived as a barrier to receiving antenatal care child vaccination services.

*“The system here (at hospital) is not on patients’ side. The doctor has quite a lot of preparation such as drinking morning tea, dressing. It takes them a lot of time to do all these before they can attend to our needs.” Female participant.*

##### Lack of beds/seats at maternity care services

In addition to waiting time, women perceived lack of space and seats in the waiting area as barriers. They had to stand during the long wait holding their infants.

Inadequate number of beds forced the local health center authorities to release patients early. Most of the men perceived that discharging the women after two hours of delivery from the local hospital was associated with post-delivery complications, such as postpartum hemorrhage, and in some cases death.

*“My wife developed complication just a few hours after she returned home (from the local health center) and I have to rush her to the private hospital because she was losing a lot of blood. I do think that the health facilities have the responsibility of discharging her too early after birth without being sure of her health.” Male participant.*

## Discussion

In Juba county in Central Equatorial State of South Sudan, our study participants experienced barriers to receive pregnancy, delivery, post delivery and neonatal care at three different levels: household, community and health system. At the household level, women were challenged by financial ability to avail the services. Community barriers included traditional and cultural believe and overall security challenges. Inadequate service availability and other health system-related barriers such as lack of infrastructure and medical amenities in the facility also had a crucial role in women’s uptake of the services.

A the household level our study revealed that long distance to the healthcare facilities coupled with sudden onset of labor hindered the women’s access to the health facility during delivery, which was similar to other studies from low-income countries [27, 28]. In Juba, pregnant women attending public health facility faced several barriers from timely access to appropriate care such as inconvenient traveling time, lack of transport and transportation cost and delivery fees in the facility. In addition, lack of welfare support and the household financial condition plays a vital role in women’s access to health services, since over 50% of the population of the country live below the poverty line [10]. Therefore, the Government of South Sudan needs to implement interventions to minimize the distance barrier by initiating collaboration with private transport sector to provide access to affordable and reliable transport services to pregnant and post-partum women. It is also essential to remove hospital delivery fees, such as bed charge, in the public facilities. In the long term it is essential to implement birthing centre at each sub district to overcome the delay in reaching health services for delivery.

At the community level, our study highlighted security concerns as a prime reason for home delivery since most of the births happened at night and the mother and her family could not travel to the health facility due to an unsafe environment. A study from conflict-affected areas in Myanmar reported similar findings [29]. In South Sudan, the ongoing inter-tribal conflict that erupted in Juba in 2013 has accelerated violent activity at night. The high incidence of violent crimes, such as murder, armed robbery and carjacking, has affected the population especially for civilians traveling alone or in small groups at night [30]. As a result, most people do not risk their life to access or seek medical care outside home at night. Therefore, measures to improve safety and security throughout the country are essential in order to improve access to medical care during the night.

In addition to household and community barriers, once the pregnant women arrived at the health facility they were further challenged by quality of care they received at the health facility. Poor quality of care, such as incorrect diagnosis at antenatal care services, lack of medicine and long waiting time was linked to dissatisfaction with services in our study, and consistent with previous findings from low-income countries [31–35]. In South Sudan, a shortage of medical doctors, midwives and nurses has severely affected service delivery in the public sector as 90% of health posts are filled with unqualified staff [18]. This situation has been exacerbated by a combination of health system failures, such as inappropriate diagnosis and treatment for pregnancy and delivery complications, poor performance of health staff, poor health service infrastructure and lack of essential medical supplies and resource [36]. Consequently, mothers and their newborn children attending such services faced poor diagnosis and treatment, which increased their risk of illness and death. The Government of South Sudan needs to invest in the human resources for health, and upgrade the exiting health services with the essential resources, supplies and training in order to improve the quality of the services. Increased budget allocations for medicine and health supplies and improving management of medicine and supply chain logistics are essential.

### Limitations of the study

Our study has some limitations that should be considered when interpreting the results. It was not possible to explore the perceptions of the women in all the health facilities in South Sudan due to the limited study budget and for security reasons. However, generalization is not the primary goal of this qualitative study. Our study samples were selected from three public health facilities in order to explore in-depth the barriers the women encountered to access and receive health services from the user’s perspectives, and capture diversity as much as possible within the scope of the study. We could not capture the perceived barriers faced by women in the rural areas due to a lack of security for data collection.

## Conclusion

This study highlighted the impact of economic and geographical accessibility, health services availability, tradition and beliefs and safety and security on access to maternal healthcare services. In order to reduce the delay in reaching health facility and to improved facility delivery, there is an urgent need for the Government of South Sudan to intervene to each of the access barriers. The Government of South Sudan needs to implement security and safety measures to ensure women’s access to services for their delivery at a facility during the night. Removing delivery fees (bed fees) in the public health centers is essential to minimize delay associated with out of pocket fees for accessing services. Upgrading the exiting health services with the essential resources, supplies and training is also essential.

## Abbreviations

MoH-GoSS: Ministry of Health, Government of South Sudan
SSP: South Sudanese pound
Trained-TBAs: Trained traditional birth attendance
USD: United States dollar
